# Developmental Role of Adenosine Kinase in the Cerebellum

**DOI:** 10.1523/ENEURO.0011-21.2021

**Published:** 2021-05-28

**Authors:** Hoda Gebril, Amir Wahba, Xiaofeng Zhou, Tho Lai, Enmar Alharfoush, Emanuel DiCicco-Bloom, Detlev Boison

**Affiliations:** 1 Department of Neurosurgery, Robert Wood Johnson Medical School, Rutgers University, Piscataway, NJ 08854; 2 Department of Neuroscience and Cell Biology/Pediatrics, Rutgers Robert Wood Johnson Medical School, Rutgers University, Piscataway, NJ 08854

**Keywords:** adenosine, adenosine kinase, cerebellum, development, cell proliferation, granule neuron precursors

## Abstract

Adenosine acts as a neuromodulator and metabolic regulator of the brain through receptor dependent and independent mechanisms. In the brain, adenosine is tightly controlled through its metabolic enzyme adenosine kinase (ADK), which exists in a cytoplasmic (ADK-S) and nuclear (ADK-L) isoform. We recently discovered that ADK-L contributes to adult hippocampal neurogenesis regulation. Although the cerebellum (CB) is a highly plastic brain area with a delayed developmental trajectory, little is known about the role of ADK. Here, we investigated the developmental profile of ADK expression in C57BL/6 mice CB and assessed its role in developmental and proliferative processes. We found high levels of ADK-L during cerebellar development, which was maintained into adulthood. This pattern contrasts with that of the cerebrum, in which ADK-L expression is gradually downregulated postnatally and largely restricted to astrocytes in adulthood. Supporting a functional role in cell proliferation, we found that the ADK inhibitor 5-iodotubericine (5-ITU) reduced DNA synthesis of granular neuron precursors in a concentration-dependent manner *in vitro*. In the developing CB, immunohistochemical studies indicated ADK-L is expressed in immature Purkinje cells and granular neuron precursors, whereas in adulthood, ADK is absent from Purkinje cells, but widely expressed in mature granule neurons and their molecular layer (ML) processes. Furthermore, ADK-L is expressed in developing and mature Bergmann glia in the Purkinje cell layer, and in astrocytes in major cerebellar cortical layers. Together, our data demonstrate an association between neuronal ADK expression and developmental processes of the CB, which supports a functional role of ADK-L in the plasticity of the CB.

## Significance Statement

The role through which adenosine metabolism functions in the developing and adult cerebellum (CB) is poorly understood. Here, we investigated the expression and possible function of adenosine kinase (ADK) during CB development. We report that ADK-L expression is associated with cerebellar development and linked to neural progenitor cell proliferation *in vitro*. In contrast to cerebrum, the adult CB maintained high levels of both isoforms of ADK (L and S).

## Introduction

The purine ribonucleoside adenosine affects brain function through adenosine receptor dependent as well as through epigenetic mechanisms (Boison, 2009, 2013). Dysregulation of adenosine is not only implicated in a wide range of brain pathologies including epilepsy, and neurodegeneration, but also in developmental pathologies (Boison, 2008; Boison et al., 2012; Masino et al., 2013; Boison and Aronica, 2015). In the brain, adenosine levels are largely under the control of adenosine kinase (ADK), the key metabolic enzyme for adenosine, which exists in a short cytoplasmic isoform ADK-S and a long nuclear isoform ADK-L (Boison, 2013; Boison and Yegutkin, 2019). Several lines of evidence show a tight association of dynamic ADK expression changes with developmental processes of the cerebrum. During prenatal and postnatal brain development there is a coordinated shift of ADK expression from nuclear ADK-L to cytoplasmic ADK-S and from neurons to astrocytes (Studer et al., 2006; Kiese et al., 2016; Gebril et al., 2020). In the adult cerebrum ADK-L expression is maintained only in astrocytes and in neurons of neurogenic areas, such as in neurons of the olfactory bulb and in dentate granular neurons of the hippocampal formation (Gouder et al., 2004; Studer et al., 2006). We recently provided evidence that ADK plays a hitherto unrecognized role in the regulation of hippocampal neurogenesis after a traumatic brain injury (Gebril et al., 2020). Together those findings support a role of ADK, and in particular of ADK-L that has epigenetic functions (Boison, 2013; Boison and Yegutkin, 2019), in developmental processes of the brain. Whereas the role of ADK in the forebrain has been widely studied (Boison, 2013), there is a paucity of knowledge about the role of ADK in the cerebellum (CB). The CB is a unique part of the brain not only involved in the control of motor function, but also plays a role in learning, cognition, and emotional functions. Cerebellar dysfunction results in several neurologic pathologies ranging from cerebellar ataxia to psychiatric conditions such as cerebellar cognitive affective syndrome (Schmahmann, 2004; Wagner et al., 2017; Nalivaeva et al., 2018). The CB differs in several important aspects from the cerebrum:

(1) Development. The CB continues to develop after birth and undergoes several cytoarchitectural changes until adulthood (ten Donkelaar et al., 2003; Rossman and DiCicco-Bloom, 2008; DiCicco-Bloom and Obiorah, 2017). It therefore offers a window of opportunity to study developmental processes in a part of the postnatal and adolescent brain. During a protracted developmental trajectory, the CB undergoes dramatic changes and morphogenesis that requires coordination of several mechanisms, including mitosis, apoptosis, cell fate determination, migration, synaptogenesis, and differentiation. In mammals, the embryonic and early postnatal CB maintains an external granular layer (EGL) of proliferative granular neuronal precursors (GNPs; Rossman and DiCicco-Bloom, 2008). As the CB develops, this layer of proliferative cells migrates radially inward to form an internal granular layer (IGL) of fully differentiated granular neurons where they integrate into cerebellar circuitry. These coordinated changes during GNPs development are guided by cellular cues from adjacent neurons located in the Purkinje layer (PL; DiCicco-Bloom and Obiorah, 2017). Because of the protracted development of the CB which extended postnatally, we hypothesized that ADK-L expression patterns during cerebellar development would support a link to developmental processes.

(2) Cell-type specificity of adenosine regulation. Whereas the cerebrum has a high abundance of astrocytes with the astrocyte to neuron ratio varying between 4:1 and 10:1, the CB is neuron rich with an inverted astrocyte to neuron ratio of 1:4 (Azevedo et al., 2009; DiCicco-Bloom and Obiorah, 2017). The adenosine tone in the cerebrum is largely controlled by metabolism through astrocytic ADK-S (Etherington et al., 2009). However, although the astrocyte to neuron ratio in the CB is low, ADK is also the primary determinant of the adenosine tone in the CB (Wall et al., 2007). Therefore, we asked whether ADK was indeed expressed in the major neuronal populations of the CB.

In the developing CB, mechanisms of adenosine release and clearance are poorly understood (Atterbury and Wall, 2009). Because ADK plays a role in development, plasticity, and cell proliferation (Boison, 2013; Boison and Yegutkin, 2019), the goal of this study was to provide a thorough understanding of ADK expression during cerebellar development in mice.

## Materials and Methods

### Animals

All animal procedures were conducted in an Association for Assessment and Accreditation of Laboratory Animal Care (AALAC)-accredited facility in accordance with approved institutional animal care and use committee (IACUC) protocols and the principles outlined in the National Institutes of Health *Guide for the Care and Use of Laboratory Animals*. All mice were on the C57BL/6 background and were socially housed under standardized conditions of light, temperature and humidity, environmental enrichment and had access to food and water *ad libitum*. Sex of prenatal, embryonic, and postnatal mice was not specified while all adult mice used for this study were males.

### Western blottings

Brains were extracted at different embryonic (E) and postnatal (P) developmental stages (E5, E10, E15, E20, P0, P1, P2, P3, P5, P7, P9, P14, P15, P20, P21, and adult) from C57BL/6J mice (total *n* = 47). Total CB was immediately dissected and frozen in liquid nitrogen vapor, then stored at −80°C. Brain samples were homogenized in RIPA buffer containing protease inhibitors (Sigma-Aldrich). Protein content was assessed using a Thermo Fisher Scientific BCA Protein assay kit. For electrophoresis, 20 μg of aqueous protein extracts were loaded and separated on 10% SDS-PAGE gels and transferred to polyvinylidene difluoride (PVDF) membranes (Bio-Rad). The blots were probed overnight at 4°C in Tris-buffered saline (TBS; 20 mm Tris, 150 mm NaCl, and 0.1% Tween, pH 7.5) containing 3% non-fat dry milk, and polyclonal rabbit anti-ADK (Bethyl Labs, A304-280A, 1:4500). The membranes were washed in TBS, and then incubated in TBS containing 5% non-fat dry milk, 1% BSA and goat anti-rabbit secondary antibody (Thermo Fisher Scientific, G-21 234, 1:10,000). Immunoreactivity (IR) was scanned and digitized using Invitrogen iBrightCL1500 Imaging System. Bands of ADK-L and ADK-S were quantitatified using ImageJ V. 1.52 software and expressed as optical densities of β-tubulin-normalized bands. All values are presented as mean ± SEM (*n* = 3–10 mice per age group). One-way ANOVA with Tukey’s multiple comparison *post hoc* test (*p ≤* *0.05, **p ≤* *0.01, ***p ≤* *0.001, ****p ≤* *0.0001 for significance).

### Immunohistochemistry

Mice at the age of P0, P2, P5, P9, P15, P21 (P0–P21) as well as adult mice were anesthetized and transcardially perfused with ice cold 4% paraformaldehyde. Brains were postfixed for 24 h in 4% PFA. Following postfixation, brains were transferred to 30% sucrose with 0.1% sodium azide in 1 × PBS for 2 d at 4°C and stored at –80°C. Brains were then cut sagittally into 30-μm sections on a freezing, sliding stage cryostat (Leica CM3050S). Until further processing, all adult brain sections were stored in cryoprotectant. Immunohistochemistry of mouse brain was performed on free-floating sections. For postnatal tissues P0–P21, sagittal 30-μm sections were directly mounted on slides then frozen until further processing. For immunofluorescence staining, antigen retrieval was performed using sodium citrate buffer pH 6 at 90°C for 3 min, then sections were washed in 1× TBS and 0.05% Triton X-100 (TBS-T) three to four times. Then, sections were incubated at 4°C overnight in donkey blocking buffer (DBB) containing corresponding primary antibodies. The primary antibodies used were, polyclonal goat anti-calbindin (Cal; Abcam ab156812, 1:250), polyclonal rabbit anti-ADK (Bethyl Labs, A304-280A, 1:1000), monoclonal mouse anti-glial fibrillary acidic protein (GFAP; Thermo Fisher Scientific, 14-9892-82, 1:1000) and monoclonal rat anti-Ki67 (Thermo Fisher Scientific, 14-5698-82, 1:200). Sections were washed in 1× TBS-T, incubated for 1 h at room temperature in a solution containing the corresponding secondary antibodies. Secondary antibodies included donkey Alexa Fluor 488 (Invitrogen, A21208, 1:2000), donkey Alexa Fluor 555 (Thermo Fisher Scientific, A-21432, 1:1000), and donkey Alexa Fluor 633 (Invitrogen A21082, 1:250). Sections were washed three times for 5 min in 1× TBS, then mounted on slides and allowed to dry in the dark. Once dried, sections were cover-slipped with DAPI mounting medium and stored in the dark at 4°C.

For 3,3′-diaminobenzidine (DAB) staining, antigen retrieval was performed using sodium citrate buffer pH 6 at 95°C for 3 min, then brain sections were washed five times for 5 min in PBS-T, then quenched in 0.3% H_2_O_2_ for 30 min. Sections were then washed three times in PBS-T at room temperature, then blocked for 1 h in goat blocking buffer (GBB). Sections were then incubated overnight at 4°C in GBB containing the primary antibody, polyclonal rabbit anti-ADK (Bethyl Labs, A304-280A, 1:1000). Sections were washed three times with TBS and then incubated in GBB containing biotinylated goat anti-rabbit IgG (1:5000) secondary antibody. After three washes in PBS-T, sections were incubated in avidin-biotin horseradish peroxidase complex solution, then in DAB substrate solution (Vector Laboratories, SK-4105) for up to 10 min until the reaction product visualized. For the slide-mounted premature tissue, the time for the reaction is longer than that of free-floating mature tissue. To standardize the experimental condition, tissues (either postnatal or adult) were exposed to the same sectioning, immunohistochemistry, and imaging conditions. Standardization was maintained between groups/tissues exposed to the same sectioning and treatment conditions.

Sections were washed three times for 5 min in PBS-T then mounted on slides and allowed to dry. Sections were dehydrated in alcohol, cleared in xylene, and mounted with mounting medium (Fisher Scientific, SP15-100 UN1294).

### Image acquisition and cell counting

Diaminobenzidine and fluorescence images were acquired using a Leica microscope fitted with a StereoInvestigator system (Microbrightfield) comprising color and monochrome digital cameras. For image analysis, we selected four 30-μm sections for each stain from each mouse, spaced every 150 μm, and spanning the same mid-lateral location between animals (*n* = 3–4 animals per group). An unbiased exclusion of poor quality images was made before analysis. Cell counting was performed by an individual blinded to the age of the animals using ImageJ software (ImageJ, National Institutes of Health; http://imagej.nih.gov/ij/). Double labeled cells in the EGL (Ki67/ADK) and PL (Cal/ADK) were counted at 40× at different focal planes in Z stacked images in *n* = 3/group. Cell counts were quantified in at least two to three sections per animal as cell number per mm^2^. Binarization of Cal, Ki67-stained, and ADK-stained images was performed using the ImageJ software Auto Threshold command. The optimal threshold values for each stain were achieved by manually adjusting the range of pixel intensity on a set of images (Crews et al., 2006). To maintain consistentcy throughout all sections, the determined range of pixel intensities was then applied for the rest of images. Using the “cell counter” command, the total number of cells in the region of intererest was determined.

### Cell culture and proliferation assay

P7–P9 mice were rapidly decapitated to isolate cerebellar GNPs as previously described (Rossman et al., 2014; Nicot et al., 2002). Briefly, following removal of the skull and meninges, and horizontal transection of the dorsal cortex from the deep cerebellar tissues and nuclei, cerebella from four to six pups were incubated in trypsin-DNase solution (1% trypsin, 0.1% DNase, Worthington) for 3 min. After removing enzyme, the tissues were dissociated in DNase solution (0.05% in DMEM) by trituration in a series of fire-polished Pasteur pipettes of decreasing diameter. The dissociated cells were then pelleted by centrifugation and filtered (30-um nylon mesh; Tekton) to remove clumps. Cells were then resuspended and centrifuged at 3200 rpm on a Percoll (Sigma) 35:60% step gradient. We collected cells at the 35:60% interface and washed them in phosphate buffer. Then cells were plated at 5 × 10^6^ density onto poly-D-lysine coated (0.1 mg/ml), 24 multiwells in medium consisting of Neurobasal, 2% B27 supplement, 0.1% BSA, 50 U/ml penicillin, and 50 μg/ml streptomycin. Cultures were maintained in a humidified 5% CO_2_/air incubator at 37°C for 24 h. For thymidine incorporation studies, cells were cultured in DMSO vehicle alone or with the ADK inhibitor, 5-iodotubericine (5-ITU), over the 0–3 μm concentration range. To estimate cell proliferation, we measured DNA synthesis by using [3H]-thymidine incorporation as a marker. GNPs were plated at 100,000 cells per well in 24-well plates and incubated for 24 h. A final concentration of 1 μCi/ml [3H]thymidine (GE Healthcare) was added to 24-well plates 4 h before cell harvesting. Following aspiration of radiotracer-containing medium, cells were lifted with trypsin-EDTA solution and collected onto filterpaper using a semiautomatic cell harvester (Skatron). Incorporation of radioactive tracer was measured in the presence of luminating solution Eco-Lite (MP Biomedicals) by scintillation spectrophotometry.

### Statistical analysis

The data were analyzed using Graphpad Prism software, version 8.4.3. All data, unless specified, were presented as mean ± SEM using one-way ANOVA followed by Tukey’s multiple comparison *post hoc* test (*p ≤* *0.05, **p ≤* *0.01, ***p ≤* *0.001, ****p ≤* *0.0001 for significance).

## Results

### ADK expression in the CB

**Figure 1.**
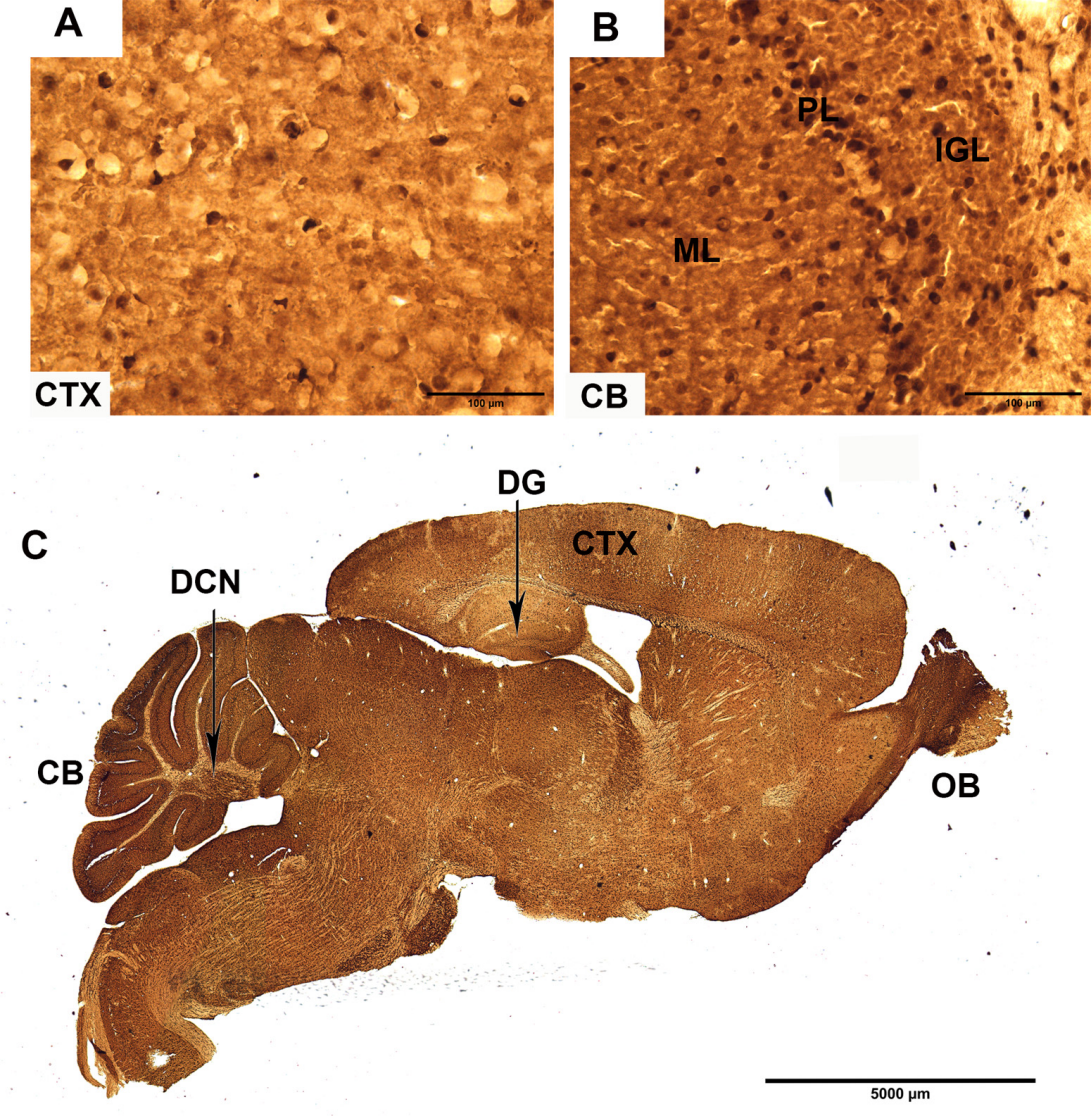
ADK IR in adult mouse brain. ADK IR as shown by peroxidase immunohistochemistry in adult mouse brain (***A–C***). ADK IR is strongly prominent in two main areas in the cerebral cortex (CTX): olfactory bulb (OB) and dentate gyrus (DG). ***A***, ***B***, In contrast to the relatively uniform staining in the CTX, ADK IR appears higher in the CB, especially in the IGL, PL, scattered cells in white matter (WM), and in deep cerebellar nuclei (DCN). The rest of the brain, including CTX and the molecular layer (ML) of cerebellar cortex is characterized by ubiquitous expression of ADK signal. Scale bars: 100 μm (***A***, ***B***) and 5000 μm (***C***).

In the adult cerebrum both forms of ADK are predominantly expressed in astrocytes (Gouder et al., 2004; Studer et al., 2006; Kiese et al., 2016) and contribute to the regulation of the tissue tone of adenosine (Etherington et al., 2009). In addition to its predominant astrocytic expression in the cerebrum, ADK-L is expressed in a limited number of neurons in neurogenic proliferative areas including olfactory bulb and dentate gyrus indicating a role in brain development and plasticity (Gouder et al., 2004; Studer et al., 2006; Boison, 2012; Gebril et al., 2020). Indeed, during the development of the cerebrum there is a coordinated shift in the expression of ADK from neurons to astrocytes (Studer et al., 2006). The CB is one of the last structures in the human brain to mature (Susan et al., 2008). In contrast to the cerebrum, the CB is also characterized by a high neuron to astrocyte ratio of 4:1 (Azevedo et al., 2009). We therefore hypothesized that the CB, a brain area in which ADK expression has not been studied before, might be characterized by a unique expression profile of both isoforms of ADK. We first assessed ADK IR in midsagittal brain sections from adult mice (Fig. 1). In line with our previous findings (Gouder et al., 2004; Studer et al., 2006; Gebril et al., 2020), we found ubiquitous expression of ADK throughout the cerebrum with relatively uniform staining all over the soma and dendrites, as well as glia (Fig. 1*A*). The only areas with higher levels of ADK IR were the olfactory bulb and dentate granular neurons in the hippocampus. In contrast, the levels of ADK IR in the CB were more intense. While ADK expression was found throughout the cerebellar cortex, the IGL, as well as neurons of the deep cerebellar nuclei (DCNs), showed intense expression of ADK. Interestingly, ADK IR was also visible in the molecular layer (ML), which contains parallel fibers of IGL neurons and interneurons, as well as glia.

### ADK expression changes during brain development

**Figure 2.**
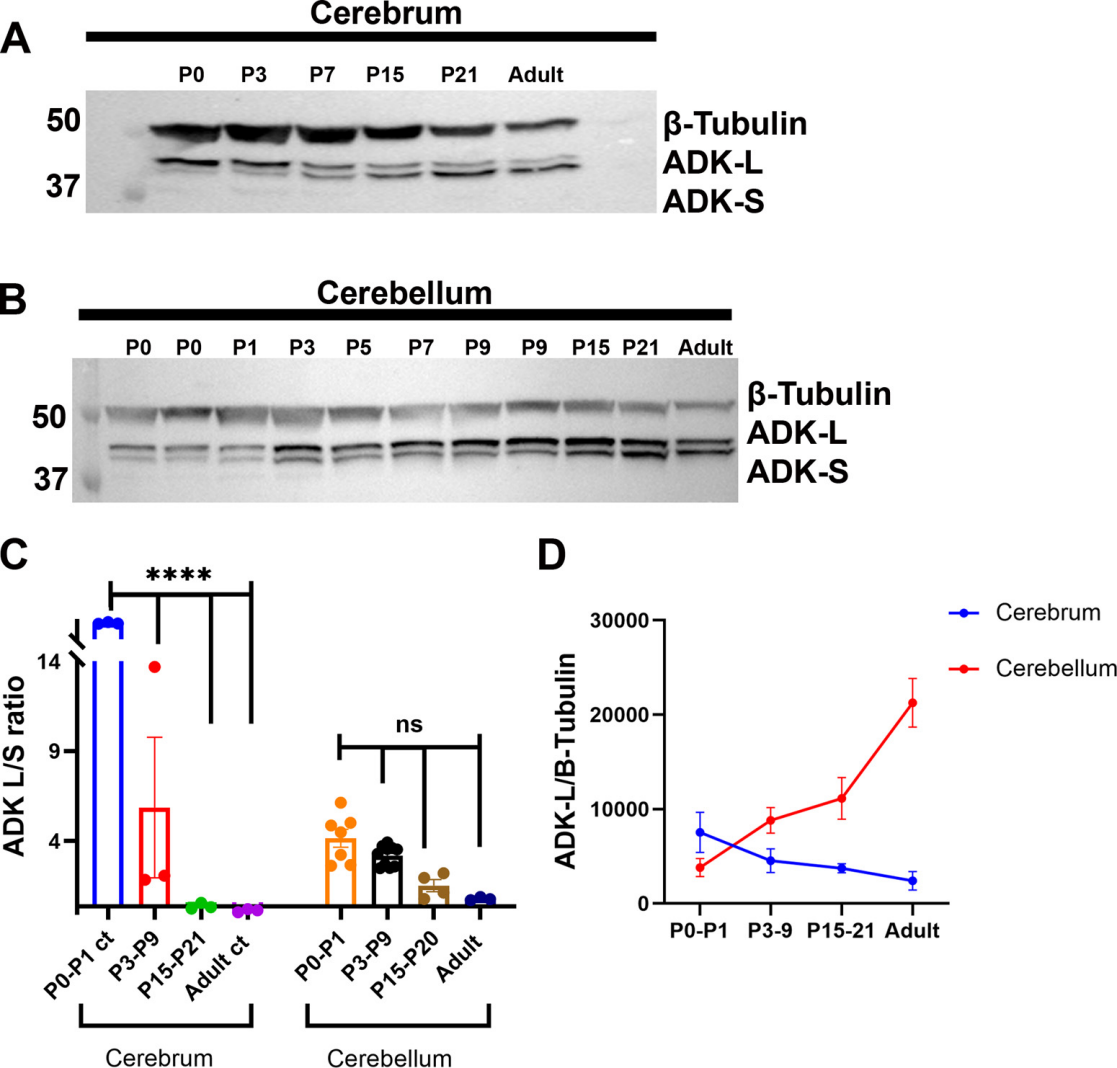
Characterization of the expression profile of ADK-L and ADK-S proteins in the developing and adult brain. ***A***, Expression profile of ADK-L and ADK-S proteins in the embryonic brain as well as the developing cerebrum. Western blot analysis shows ADK expression changes during prenatal and postnatal development of the cerebrum in the mouse. Representative blots show ADK (L and S) isoform expression at different embryonic (E) and postnatal (P) stages with ADK-L shown as the upper band and ADK-S as the lower band. In embryonic brain, the nuclear long form ADK-L dominates and shifts toward ADK-S dominance in the adult cerebrum. Control includes recombinant protein ADK-S (Rec). ***B***, Western blot analysis of the expression profile of ADK-L and ADK-S proteins in the developing and adult CB. The postnatal as well as adult CB exhibited strong expression of ADK-L while ADK-S expression increased progressively from P3 into adulthood. ***C***, Quantitative analysis of Western blot analyses using ImageJ V. 1.52 software and expressed as the ratio of optical densities of ADK-L/ADK-S bands. All values are presented as mean ± SEM (*n* = 3–10). One-way ANOVA with Tukey’s multiple comparison *post hoc* test (ns, no significance, and ****p < 0.0001 for significance). ***D***, Line graph of normalized optical density of ADK-L bands at different developmental time points of cerebrum and CB.

Given the major expression differences in ADK between the cerebrum and the CB, we next assessed the profile of ADK expression during brain development by quantitative Western blot analysis (Fig. 2). In line with a developmental role of ADK-L, we found a dominance of ADK-L as compared with ADK-S (Fig. 2*A*,*C*) in early postnatal cerebrum. During postnatal development of the cerebrum, the ADK-L/ADK-S ratio continued to decline, leading to a dominance of ADK-S expression in adulthood (Fig. 2*A*,*C*). Whereas ADK-L expression is downregulated during postnatal development of the cerebrum (P0–P1 vs P3–P9, P15–P21, and adult, *p* < 0.0001), in CB its expression continued to be maintained at high levels across developmental stages and even in the mature CB (P0–P1 vs P3–P9, P15–P21, and adult CB, *p* = 0.97, *p* = 0.41, and *p* = 0.24, respectively; Fig. 2*B*,*C*). Because the expression levels of ADK-S gradually increased during postnatal development of the CB starting from P3 and continuing into adulthood, there was a gradual drop in the ADK-L/ADK-S ratio (Fig. 2*C*). Overall, this analysis demonstrates that, in contrast to the cerebrum, the dominance of ADK-L, is maintained throughout adulthood in the CB (Fig. 2*D*), suggesting that the CB maintains a distinct expression profile of ADK, raising questions about its functional role(s).

### Adenosine kinase expression is associated with development of the cerebellar cortex

**Figure 3.**
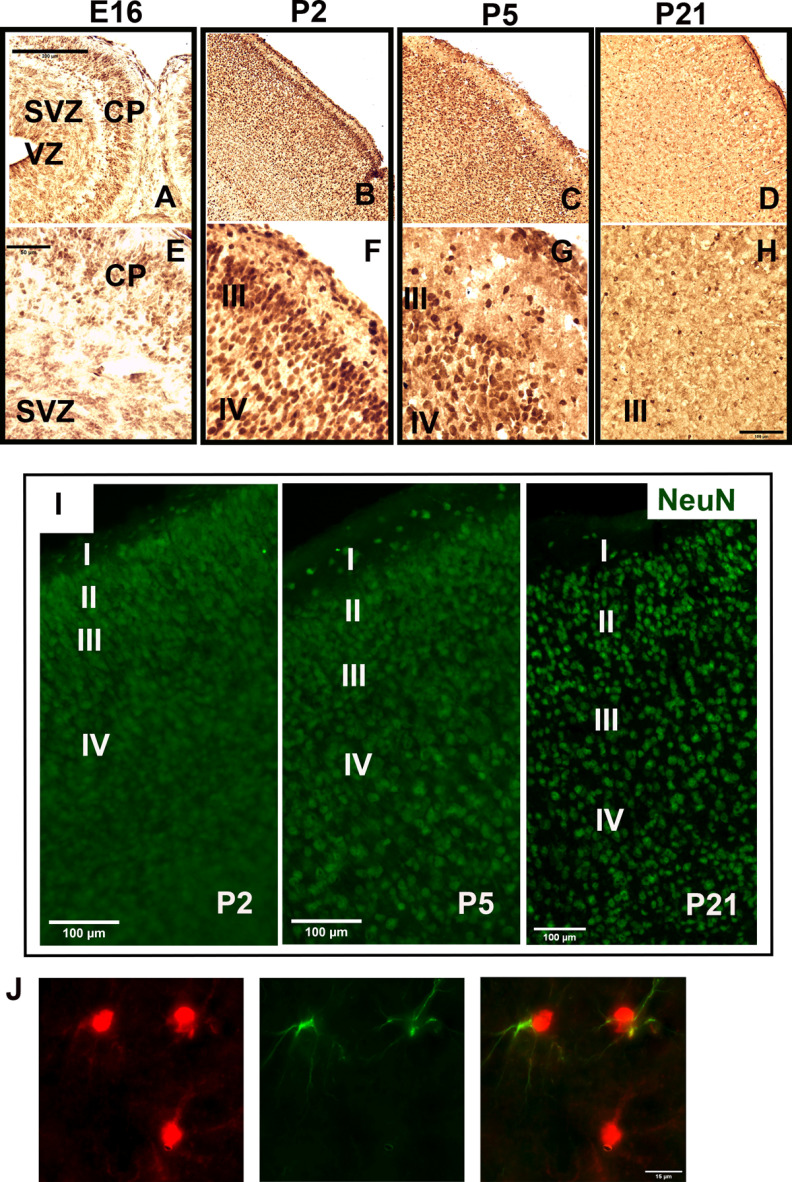
ADK IR in prenatal and postnatal mouse cerebrum. ADK IR in the mouse cerebrum at different developmental stages; E16, and postnatal days (P2, P5, and P21) as shown by peroxidase staining. ***A***, ***E***, At E16, nuclear expression of ADK presented as dark punctate staining in cells of the ventricular zone (VZ), sub-ventricular zone (SVZ), cortical plate (CP) in the neocortex. ***B***, ***F***, At P2, most of nuclear ADK IR is observed in Layers II/III/IV of the neocortex. ***C***, ***G***, At P5, nuclear ADK IR is only observed in Layers III/IV**. *D–H***, At P21, nuclear ADK IR reduced while diffuse IR consistent with cytoplasmic ADK-S is widespread. ***I***, NeuN (green) IR illustrating cortical layers as shown by immunofluorescence at developmental stages P2, P5, P21. ***J***, Single-cell image of ADK (red)-positive, GAFP (green) astrocytes in P21 cerebrum. Scale bars: 200 μm (***A–D***) 100 μm (***H***, ***I***), 50 μm (***E–G***), and 15 μm (***J***).

**Figure 4.**
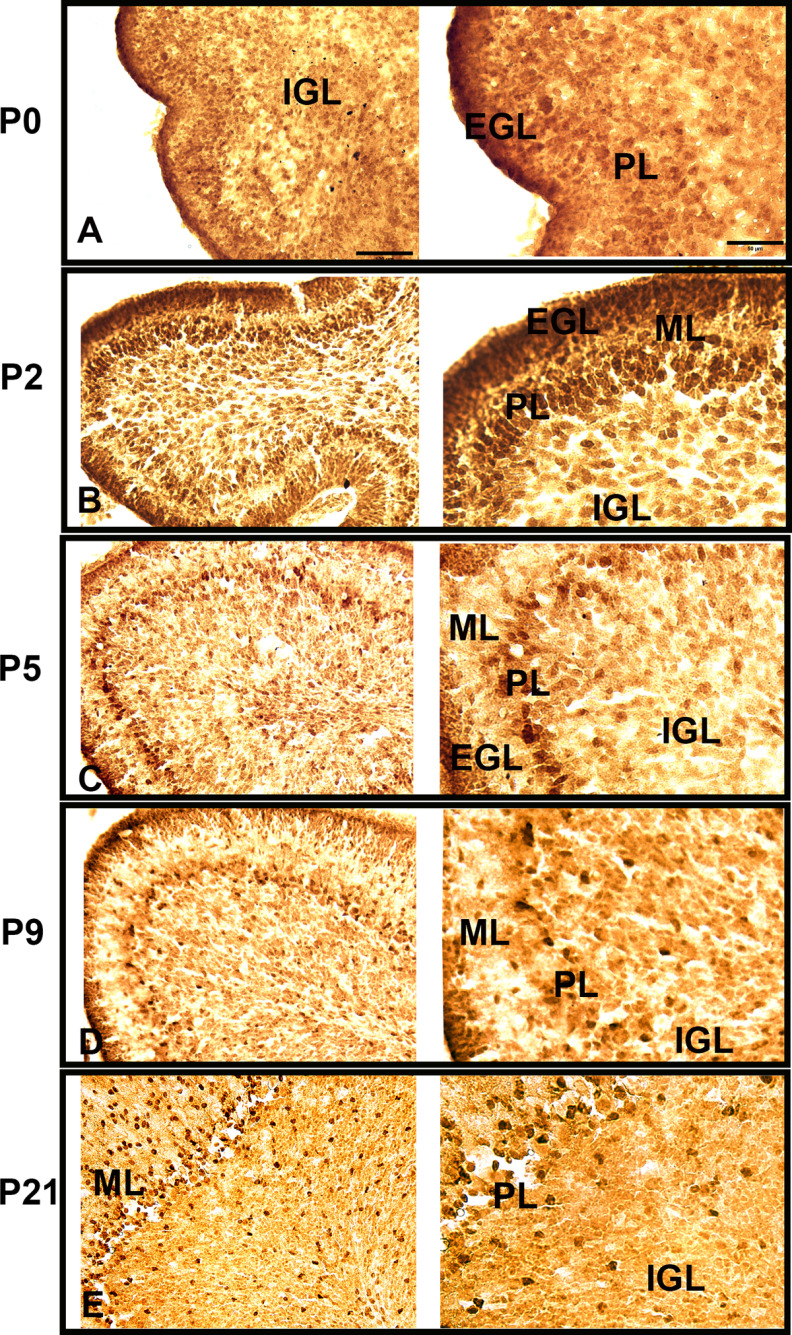
ADK IR in postnatal mouse CB. ADK IR in mouse CB at different developmental stages (P0, P2, P5, P9, and P21) as shown by peroxidase staining. ***A***, At P0, ADK IR is widespread in the external granular layer (EGL), Purkinje layer (PL), and internal granular layer (IGL). ***B***, At P2, ADK IR is present in the distinct layers of the cerebellar cortex EGL, PL, IGL, whereas fewer ADK-positive cells are observed in the molecular layer (ML). ***C***, ***D***, At P5 and P9, ADK IR declines in EGL, PL, and ML. The expression pattern of ADK IR appeared as dark punctate staining in the cells of all layers, which suggests the dominance of ADK-L at these developmental stages as seen in high magnification images in the right panel. ***E***, At P21, no ADK is detected in the outermost layer of the cerebellar cortex. The dark punctate staining consistent with ADK-L is scattered in ML, PL, and IGL, whereas the diffuse staining of ADK-S is extensively observed in ML and IGL. Scale bars: 100 μm (left panel) and 50 μm (right panel).

The Western blot analysis (Fig. 2) revealed an association of ADK-L with the development of the CB. Recent findings that ADK-L is associated with neurogenic areas in the developing and adult cerebrum (Gebril et al., 2020) were the foundation to hypothesize that ADK-L is also associated with neurogenic areas in the developing CB. To address this hypothesis, we examined and compared the pattern of ADK expression and distribution during the development of the cerebrum and CB. Because neurogenesis of the cerebrum occurs predominantly during prenatal development and declines thereafter (König and Marty, 1981; Noctor et al., 2002; Shen et al., 2006; Rakic, 2007), ADK IR was assessed during the perinatal period between E16 and P21 (Fig. 3). At E16, the ventricular zone (VZ), sub-VZ (SVZ), and cortical plate (CP) were strongly positive for ADK. According to previous work (Gouder et al., 2004; Studer et al., 2006); ADK-S expression appears as diffuse staining throughout the brain tissue, whereas ADK-L signal appears as dark punctate staining of nuclei. Therefore, the darkly stained nuclei of progenitor cells at the early developmental stages would then be consistent with expression of the nuclear isoform, ADK-L. The intense signal of ADK IR gradually declined progressively from P2 and P5, but was maintained in Layers III/IV of the neocortex (Fig. 3*B*,*C*,*F*,*G*). By P9, ADK IR continued to disappear even in Layer III of the cortex (data not shown). At P21, intense nuclear ADK IR was extensively reduced, which coincides with the maturation of pyramidal neurons in the cerebral cortex (Zhang, 2004; Shen et al., 2006; Fig. 3*D*,*H–J*). In contrast, in the CB, neurogenesis continues postnatally (Carletti and Rossi, 2008); therefore, the expression of ADK in the neurogenic zones was studied in midsagittal sections at developmental stages between P0 and P21 (Fig. 4). At P0, ADK IR was widespread in the CB, including the EGL, PL, and the emerging IGL (Fig. 4*A*). At P2, ADK IR was robust in distinct layers of the cerebellar cortex including EGL, PL, and IGL, whereas fewer ADK-positive cells were detected in the ML (Fig. 4*B*). The qualitative signal intensity of ADK-positive cells appeared to be less in the EGL at P5 and P9, which coincides with the active migration of cells from the EGL into the IGL (Carletti and Rossi, 2008; DiCicco-Bloom and Obiorah, 2017; Fig. 4*C*,*D*). There appeared to be similar reduction in ADK signal intensity in the PL and ML at P5 and P9 as compared with P2, though absolute quantification was not performed. Interestingly, the expression pattern of ADK from P0 up to P9 appeared as dark punctate staining in the nuclei of the cells in all layers and therefore supports the dominance of ADK-L over ADK-S at this developmental stage, which is in line with our Western blot analysis (Fig. 2). At P21, as the EGL disappeared, the pattern of ADK staining has shifted to the outermost layer of the cerebellar cortex, the mature ML that contains axons of the IGL neurons (Fig. 4*E*). The dark punctate staining consistent with ADK-L was scattered in ML, PL, and IGL, whereas the diffuse staining of ADK-S was extensively observed in ML and IGL of P9 and P21. This pattern is in line with the Western blotting (Fig. 2*B*) results, which confirmed the strong expression of both isoforms L and S at these developmental stages. We tentatively conclude that the expression of ADK-L is associated with neurogenic areas in the cerebrum and the CB. This finding suggests a role of ADK in the growth and development of both brain regions. In contrast to the cerebrum however, ADK-L expression is maintained in mature neurons of the IGL, which suggests a specific role of ADK-L in the mature CB.

### ADK-L is associated with the development of cerebellar granule neurons

**Figure 5.**
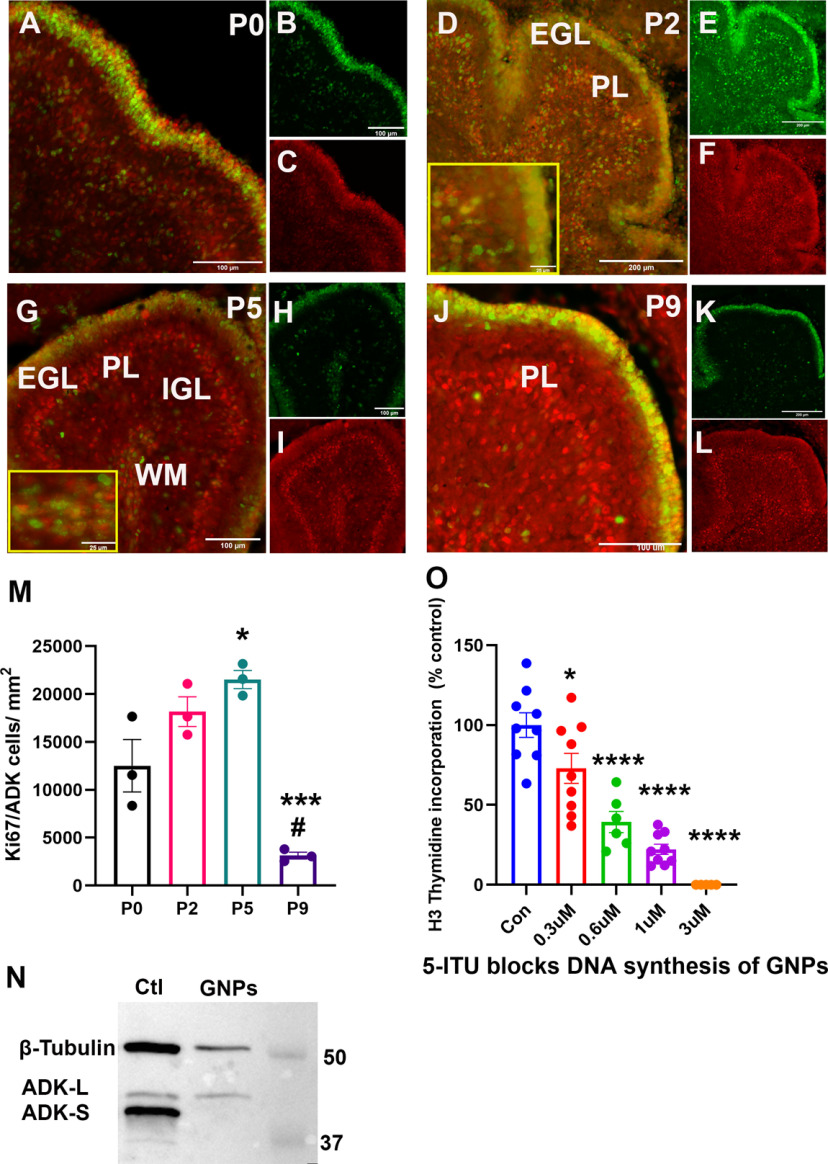
Effect of ADK on DNA synthesis in the developing CB. ***A***, ADK (red) and Ki67 (green) IR in the mouse CB at different developmental stages (P0, P2, P5, and P9) as revealed by double immunofluorescence. ***A–C***, At P0, ADK/Ki67 double-labeled cells are widespread in the entire developing cerebellar cortex with robust expression at the outermost EGL. ***D–F***, At P2, the layers of cerebellar cortex become visible where ADK/Ki67-positive cells are observed in the external granular layer (EGL), the developing Purkinje layer (PL), and the internal granular layer (IGL). ***G–L***, At P5 and P9, ADK/Ki67-labeled cells initially increase then decrease in the EGL, which period coincides with the radial migration of cerebellar granular neurons. ***G***, In the internal white matter (WM) region at P5, a population of ADK/Ki67-positive cells was observed, which declined in P9 (***H***). Scale bars: 100 μm (***A–C***, ***G–L***), 200 μm (***D*–*F***), 25 μm (***D***, ***G***, insets). ***M***, The number of Ki67/ADK double labeled cells in the EGL at P0, P2, P5, and P9 per mm^2^ (*n* = 3/group). One-way ANOVA with Tukey’s multiple comparisons *post hoc* test (*p < 0.05 for P5 vs P0, ^#^*p* < 0.05 for P9 vs P0, and ***p < 0.001 for significance). *N*, Western blot analysis of immature granular neuronal precursors (GNPs). Representative blot of protein extracts from control (Ctl) glioblastoma U373 cells and immature GNPs shows that the precursors express ADK-L exclusively whereas control cells express both isoforms. ***O***, Inhibition of ADK with antagonist 5-iodotubercidin (5-ITU) reduces DNA synthesis of GNPs. Concentration-dependent reduction of cell proliferation in response to different concentrations (0.3, 0.6, 1 μm) of the ADK inhibitor 5-ITU as compared with vehicle (DMSO ≤ 0.002%)-treated cells. All values are presented as mean ± SEM (*n* = 6–9). One-way ANOVA with Tukey’s multiple comparisons *post hoc* test (*p < 0.05, ***p < 0.001, and ****p < 0.0001, for significance).

Because ADK-L is expressed during the period of neurogenesis in the developing CB, we hypothesized that ADK-L plays roles in GNPs proliferation and postnatal development of diverse cell types. To explore the proliferative cell compartment, a co-localization analysis using antibodies directed against ADK and the proliferation marker Ki67 was performed on midsagittal sections of mouse CB from P0 to P9 (Fig. 5*A–M*). At P0, cells double-labeled for ADK and Ki67 were widespread in the entire developing CB with robust expression found in the outermost EGL. As the EGL expands because of active cell divisions (Carletti and Rossi, 2008), a thick layer of ADK-positive and Ki67-positive cells appeared at P2 and P5. At P5, the number of Ki67/ADK double labeled cells was significantly increased (*p* = 0.02) as compared with P0. From P5 to P9, the thickness of the EGL initially increased, followed by gradual reduction by P9, which temporally coincides with the radial migration of GNPs into the IGL, while the number of Ki67/ADK double labeled cells was significantly reduced as compared with P0 (*p* = 0.02) and P2 and P5 (*p* = 0.009 and *p* = 0.002, respectively). Interestingly, a new population of ADK/Ki67-positive cells in the white matter appeared on P5, presumably comprised of progenitor cells of cerebellar interneurons or glial cells (Zhang and Goldman, 1996). This population of ADK/Ki67-positive progenitor cells gradually decreased at P9 then disappeared by P21 (data not shown). By P21, all GNPs in the EGL are expected to differentiate, fully migrate, and reside in the IGL (Carletti and Rossi, 2008). Here, we found that both Ki67-positive and ADK-positive cells disappeared from the outermost layer whereas ADK expression was maintained in the IGL. These findings suggest a role of ADK-L in the maintenance of cell proliferation during CB development.

### ADK-L contributes to the regulation of GNPs proliferation

To support our contention that ADK-L is involved in GNPs development and specifically in cell proliferation, we investigated the effect of inhibition of ADK-L on DNA synthesis, a precursor to cell division, of isolated GNPs. For these studies, we employed primary cultures of GNPs isolated from P7–P9 mouse pups, a period when we have found that growth factors, such as FGF, SHH, IGF1, and PACAP, regulate cell cycle progression and the production of new neurons (Rossman et al., 2014; Nicot et al., 2002; Tao et al., 1996) when EGL precursor are most abundant. Using Western blot analysis, GNPs were found to express the nuclear ADK-L isoform exclusively (Fig. 5*N*), supporting the contention that EGL precursors (Ki67-ADK double-labeled cells; Fig. 5*A–J*) express ADK-L. To assess DNA synthesis, isolated GNPs were incubated in control vehicle containing medium or media containing different concentrations (0.3, 0.6, 1 μm) of the ADK inhibitor 5-ITU, and then assessed for 3H-thymidine incorporation at 24 h (Fig. 5*O*). Strikingly, GNPs exhibited a concentration-dependent reduction in thymidine incorporation in response to 5-ITU treatment especially at high concentrations of 5-ITU [0.3 μm (*p* = 0.047), 0.6 μm (*p* < 0.0001), and 1 μm (*p* < 0.0001)]. Importantly, the reduction in DNA synthesis suggested that fewer cells entered the S phase, as there was no reduction of cell survival except at the highest concentration (3 μm). It is worth mentioning that the concentration-dependent study represents concentrations of ITU that are far below the reported effective concentration (EC_50_ = 7.8 μm; Zhang et al., 2013). The reduction in DNA synthesis following inhibition of ADK-L suggests that adenosine plays a functional role in proliferation of GNPs during cerebellar development.

### ADK-L is involved in the development and maintenance of Purkinje cells

**Figure 6.**
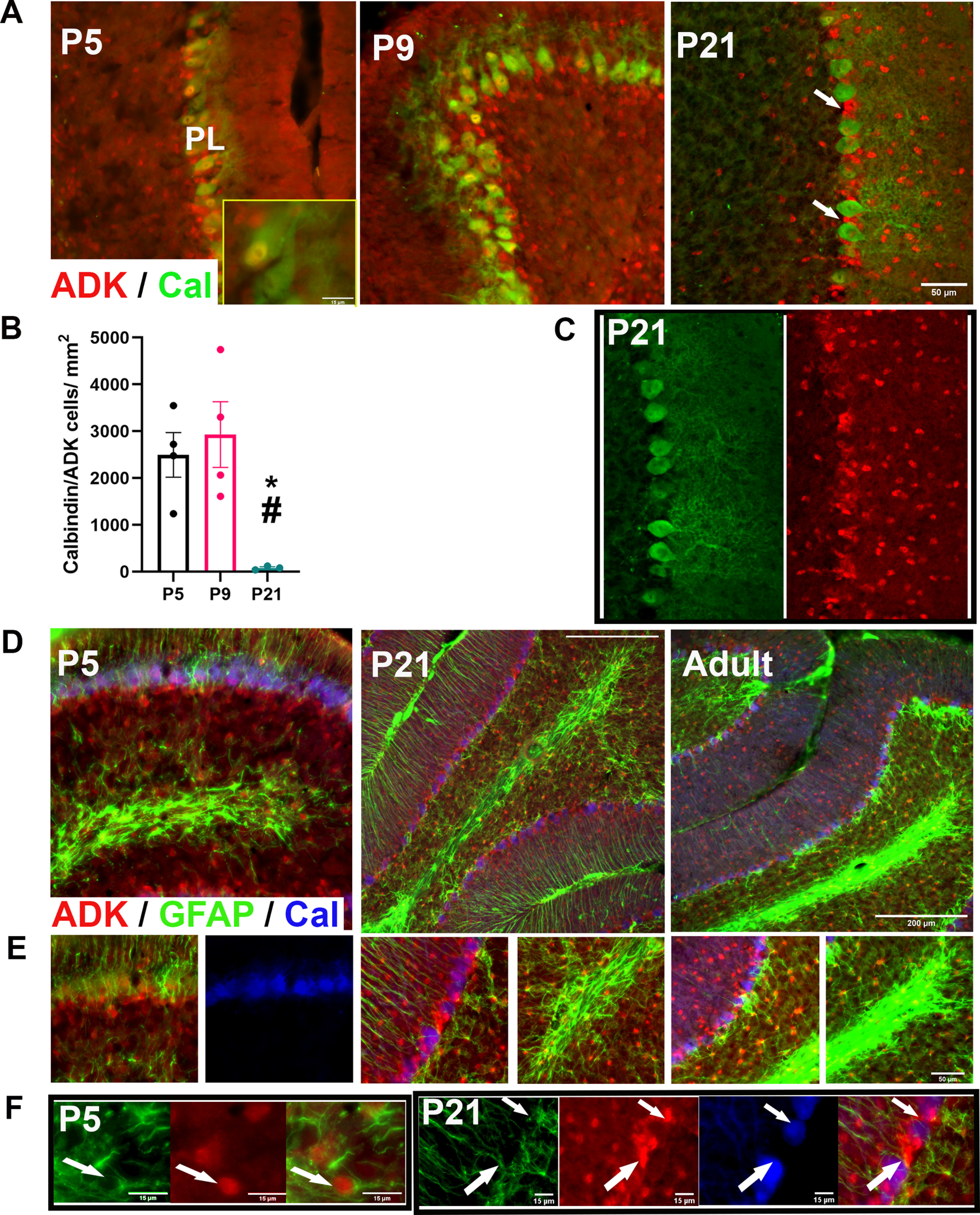
Association of ADK with the development and maintenance of cerebellar cortex cells. ***A***, ***B***, ADK (red) and Cal (green) IR in the developing cerebellum (CB) at different developmental stages (P5, P9, and P21) as shown by immunofluorescence. ***A–C***, Purkinje cells that are ADK (red)/Cal (green)-positive are observed in the Purkinje layer (PL) where the punctate stain of nuclear ADK-L is prominent at P5 and P9. By P21, ADK-L IR is less prominent in Purkinje cells and only maintained in a few cells, whereas the rest of ADK-L cells are found in clusters between Purkinje cells in the PL. White arrows in A point at ADK-L cells between Purkinje cells in P21. ***B***, The number of Cal/ADK double labeled cells in the PL at P5, P9, and P21 per mm^2^ (*n* = 3–4/group). ***D***, IR of ADK (red)/GFAP (green) in astrocytes of the cerebellar cortex at different developmental stages (P5, P21, and adult) as shown by immunofluorescence. ***E***, Bergmann glial cells in the PL show punctate staining of nuclear ADK-L at all developmental stages (P5, P21, and adult). GFAP-positive astrocytes that are ADK-L-positive are prominent in the white matter at all developmental stages while astrocytes in the inner granular layer are observed at P21 and in adulthood. ***F***, Magnified field of glial cells and ADK-positive cells in white matter of P5 and PL of P21. White arrows showed ADK-L-positive Bergman glial cells between Purkinje neurons. Scale bars: 50 μm (***A***, ***C***, ***E***), 200 μm (***D***), and 15 μm (inset in ***A***, and ***F***). One-way ANOVA with Tukey’s multiple comparisons *post hoc* test (*p < 0.05 for P5 vs P21, and ^#^*p* < 0.05 for P9 vs P21 for significance).

Since ADK was detected in the progenitor layers of the cerebellar cortex at early developmental stages, we investigated the involvement of ADK in the development of Purkinje cells. To address this, we examined the IR of cells that are positive for both ADK and Cal, a Purkinje cell marker, in midsagittal sections at selected developmental stages (P5, P9, and P21). At P5 and P9 (Fig. 6*A*), most Purkinje cells exhibited ADK signal in the nucleus. The expression of ADK in Purkinje cell nuclei at P21, however, was significantly reduced as compared with P5 (*p* = 0.038) and P9 (*p* = 0.017; Fig. 6*B*). Instead, the ADK-L-positive cells in the PL layer at P21 were located in clusters between each Purkinje cell in the PL (Fig. 6*A–C*). Those ADK-positive cells are presumably Bergmann glial cells which are characterized by cell bodies located in the PL layer around the somata of Purkinje cells.

### Bergmann glial cells express ADK-L

In young adult mice, most of the ADK-positive cells in the PL were found in clusters between Purkinje cells suggesting they might be glia. Therefore, we next sought to determine whether ADK-positive cells in the PL were astrocytes by co-localizing ADK and GFAP (Fig. 6*D*). At early developmental stages (P0, P2), GFAP was rarely detected (data not shown), whereas at P5, GFAP was highly expressed in extended cell processes in the PL and the developing white matter (Fig. 6*D*). GFAP-positive cells appeared to be ADK-positive, where ADK cell nuclei were surrounded by GFAP labeled cell somas (Fig. 6*E*,*F*). This pattern suggests that ADK-L might play a role in the development and maintenance of Bergmann glial cells and astrocytes in the inner white matter. Interestingly, these Bergmann glia cells maintained strong expression of ADK in P21 and in adulthood. Moreover, glial cells in the white matter and the IGL also strongly expressed ADK at P21 and in adulthood, suggesting a role of ADK-L in the development and maintenance of astrocytes in these regions.

### Developing and mature cerebellar neurons maintain ADK expression

**Figure 7.**
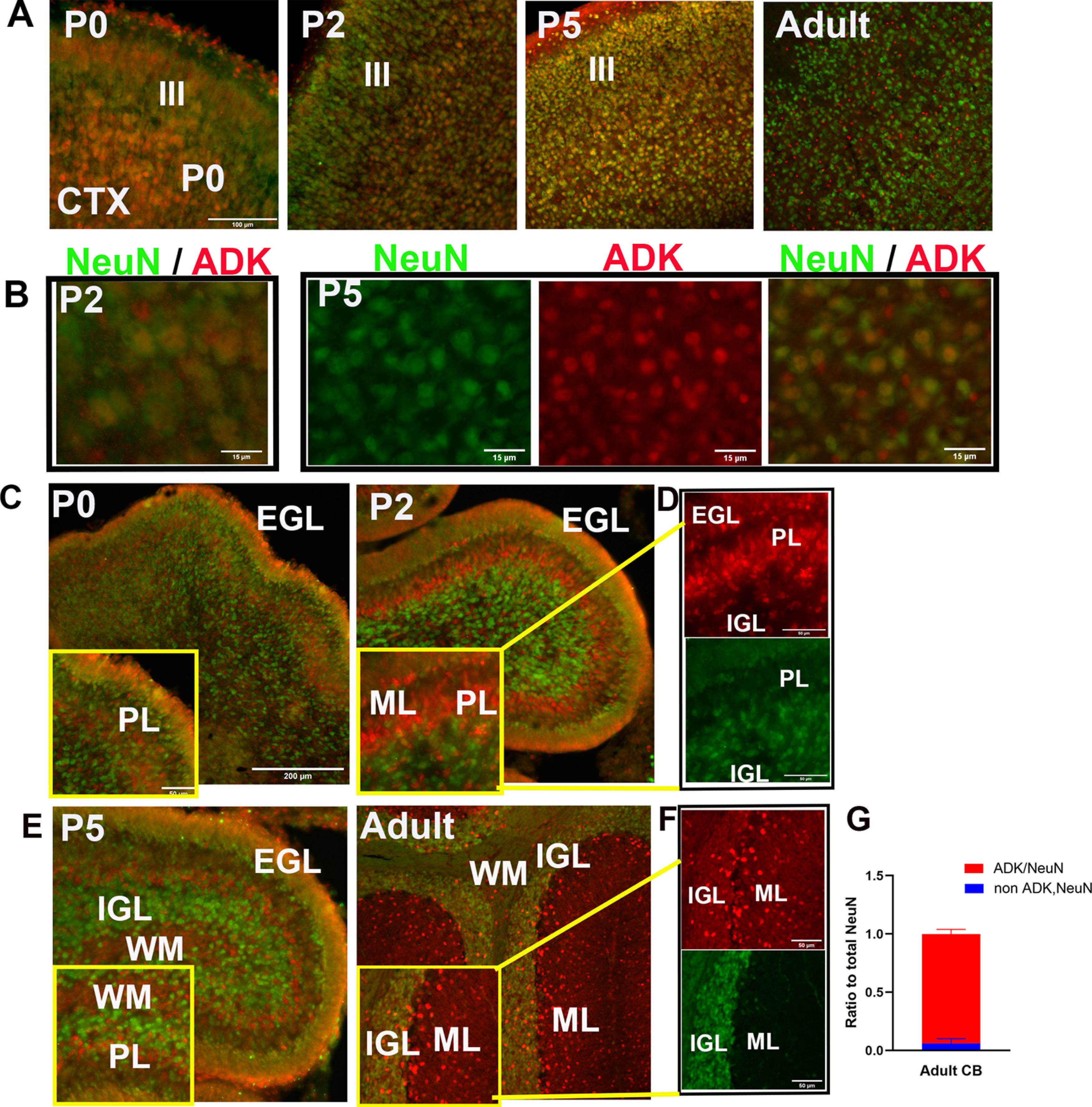
ADK expression is maintained in developing and mature cerebellar granule neurons. ***A***, Cortical neurons positive for ADK/NeuN observed in Layers III/IV of the neocortex in P0–P5, where the nuclear ADK-L stain is prominent. By P21, all NeuN cortical neurons are ADK-L-negative, while ADK expression is found in NeuN-negative cells. ***B***, Magnified fields of cortical Layer III in P0 and P5 illustrating cells positive to ADK/NeuN. ***C–F***, Cerebellar neurons positive for ADK/NeuN observed in mice from P0 to adult. Neurons in the external granular layer (EGL) and Purkinje layer (PL) are ADK-L-positive from P0 to P5 as seen in ***C–E***, insets. ***D***, ADK/NeuN-positive neurons in the innermost layer of EGL, PL, and internal granular layer (IGL) of P2. Between P2 and P5, ADK-positive neurons are detected in neurons of the PL, while positive ADK staining is maintained in cells of the EGL. ***F***, By adulthood, ADK-positive neurons are restricted only to the mature granule neurons in the IGL and their parallel fibers in the ML. Scale bars: 100 μm (***A***), 15 μm (***B***), 200 μm (***C***, ***E***), 50 μm (***D***, ***F***, and insets of ***C***, ***E***). ***G***, Ratio of NeuN/ADK-positive cells to total NeuN-positive cells in the adult IGL (*n* = 3/group).

In contrast to the cerebral cortex, the adult CB maintains considerable levels of ADK (Fig. 2), while the majority of cells are neurons (Azevedo et al., 2009). Therefore, we asked whether the developing and fully differentiated neurons of the CB express ADK, and compared the cerebral cortex and CB on midsagittal brain sections at selected developmental stages using antibodies to ADK and mature neuronal marker, NeuN. Cortical neurons in Layers III/IV of mice from P0 to P5 strongly expressed ADK-L as suggested by nuclear co-localization of ADK and NeuN (Fig. 7*A*,*B*). By adulthood, apparently NeuN-positive cortical neurons became ADK-L-negative, while ADK expression was limited to NeuN-negative cells (Fig. 7*A*). The latter cells were identified as astrocytes in previous studies (Studer et al., 2006) and supported by double labeling in Figure 3*J*. The same NeuN/ADK co-localization study was performed in the CB. At P0, cerebellar neurons of the innermost layer of EGL were ADK-L-positive, based on GNP exclusive expression of ADK-L (Fig. 5*N*), and exhibit nuclear NeuN co-localized with ADK. At P2, the ADK/NeuN co-localization in the innermost layer of EGL was maintained (Fig. 7*C*,*D*). At P2 and P5, as the newly born cerebellar neurons continue to differentiate and migrate (Carletti and Rossi, 2008), EGL neurons remain ADK-positive. In contrast in the PL, cells that are NeuN-negative exhibit strong ADK signal (Fig. 7*C*,*D*). Those cells are presumably developing Purkinje cells or glial cells. It is worth noting that ADK/NeuN-positive neurons were detected in the ML (Fig. 7*C*,*D*) reflecting neuronal migration from EGL into the IGL, or locally resident GABAergic basket and stellate neurons (Brown et al., 2019). At P5, the developing white matter, which lacks NeuN-positive neurons, exhibits widespread expression of ADK presumably a combination of interneuron progenitors and newborn glial cells. By adulthood, ADK/NeuN-positive neurons were limited to the IGL where some of these neurons are presumably interneurons according to their morphology and location (Fig. 7*E*,*F*). The vast majority of IGL neurons are ADK/NeuN-positive (Fig. 7*G*), although only a subset express intense nuclear signal, whereas the majority of NeuN staining cells exhibit more diffuse, lower intensity ADK-S signal. Those mature cerebellar granule neurons of IGL are suggested to express both forms of ADK (L and S) because of the co-localization pattern of the nuclear NeuN with ADK, whereas the parallel fibers maintain the cytoplasmic diffused appearance of ADK-S.

## Discussion

In this study, we explored a role for ADK in the developing and adult CB. First, we show that in contrast to the cerebrum, high expression levels of both ADK isoforms (L and S) are maintained in the adult CB. The expression profile of ADK-L is high throughout all cerebellar developmental stages, whereas ADK-S expression gradually increases with age. Second, we demonstrated that ADK-L is associated with proliferative progenitors in the developing CB and maintained in developing Purkinje cells. Third, ADK-L is associated with developing and mature Bergmann glial cells and astrocytes in the cerebellar cortex. Finally, ADK-L is highly expressed in mature neurons of the EGL and PL at different developmental stages while mature granule neurons in the IGL maintain strong expression of both forms of ADK in the adult CB.

### ADK plays a conserved role in brain development

In line with previous findings suggesting a functional role of ADK during both human and murine brain development (Studer et al., 2006; Gebril et al., 2020), we find similarities in the ADK expression profile during the development of both CB and cerebrum. The coordinated developmental changes of the ADK expression profile in the cerebrum (Fig. 2) is in line with the reported developmental downregulation of neuronal ADK-L transcripts and upregulation of astrocytic ADK-S transcripts during the first postnatal weeks (Kiese et al., 2016). Higher levels of ADK expression during energy demanding developmental processes may provide (1) a salvage pathway to use adenosine to generate ATP for RNA synthesis; and (2) epigenetic control of neurogenic genes necessary for neuronal proliferation and plasticity. A developmental role of ADK-L is also supported by developmental defects associated with inborn human ADK deficiency, which leads to growth defects, intellectual disability, and hepatic encephalopathy (Bjursell et al., 2011; Staufner et al., 2016; Silva et al., 2020). Likewise, the genetic deletion of ADK in mice or plants leads to striking similarities in transmethylation defects and stunted growth (Boison et al., 2002; Moffatt et al., 2002). These data as well as our recent data supporting a role of ADK-L in neurogenesis (Gebril et al., 2020) suggest that ADK-L plays a role in the development of the CB and cerebrum. Indeed, intricate similarities in the coordinated changes of the ADK expression profile shared during the development of cerebrum and CB support the idea that ADK-L plays a conserved role in cell plasticity and brain development (Studer et al., 2006; Gebril et al., 2020).

### ADK plays a role in cell proliferation and morphogenesis

The developing brain is characterized by dynamic and coordinated changes, including cell proliferation, differentiation, and migration. The expression of ADK-L is associated with the most plastic neurogenic areas in the developing as well as the adult brain (Studer et al., 2006; Gebril et al., 2020). Here, we report that in contrast to the cerebrum, ADK-L is maintained at high levels in neurons of the adult CB and is associated with cerebellar development. During postnatal development of the CB, most of the cells in the cerebellar cortex exit the cell cycle, while undergoing morphologic changes and migration toward the IGL. In this study, we found strong expression levels of ADK-L in proliferative cerebellar granular neurons of the EGL (Fig. 5) and in Purkinje cells (Fig. 6). This pattern suggests a functional role of ADK-L in maintaining cell plasticity, morphogenesis, and proliferative status as the expression of ADK-L coincides with cell proliferation and morphogenesis at early developmental stages. The developmental role of ADK-L is further supported by the concentration-dependent reduction of cell proliferation in response to pharmacological inhibition of ADK in immature GNPs primary cultures (Fig. 5). This result is in line with the recent findings that the genetic deletion of ADK-L in neurogenic areas, as well as the pharmacological inhibition of ADK, modifies baseline cell proliferation status in the neurogenic dentate gyrus (Gebril et al., 2020).

### Role of ADK-L in adult CB

The finding that ADK-L is gradually decreased as Purkinje cells mature (Fig. 7) is in line with the notion that Purkinje cells contain high levels of adenosine and adenosine receptors (Goodman et al., 1983; Braas et al., 1986; Namba et al., 2010). Whereas in cerebellar granule neurons, except their excitatory axons and neurons of deep nuclei, adenosine receptors are undetectable (Goodman et al., 1983; Kocsis et al., 1984; Namba et al., 2010). This supports our findings that ADK expression is high in both developing and mature cerebellar granule neurons.

It is well established that developing Purkinje cells are essential for the proliferation and differentiation of afferent neurons, especially cerebellar granule neurons, while they become dependent on signals from mature cerebellar granule neurons (Behesti and Marino, 2009). Our data suggest the involvement of ADK-L in this reciprocal signaling and circuitry in the CB, because ADK-L is associated with cells that provide signaling cues and guidance, such as developing Purkinje cells and mature cerebellar granule neurons. Given that the neuron to astrocyte ratio is high in the CB (Azevedo et al., 2009), neuronal adenosine metabolism might not only be essential for cell signaling but also to establish metabolic homeostasis and to support the high energy demand for cerebellar neurons. This implies a metabolic role of ADK in the mature CB.

In this study, we provide evidence that ADK plays a conserved role during brain development given the similarities of ADK expression profiles in the developing CB and cerebrum. Here, we elucidated the spatio-temporal and cell-type specific ADK expression profile in the developing CB. Based on our findings we suggest two critical functions of ADK in the developing CB, first ADK may work as a salvage pathway enzyme to power anabolic reactions important for cell development. Second, ADK may work as an epigenetic regulator of neurogenic genes important for neuronal proliferation and plasticity. In contrast to the cerebrum, the neuron to astrocyte ratio is high in the CB; therefore, metabolic support provided to cortical neurons by ADK expression in cerebral astrocytes may be alternatively provided by resident neurons in the CB in the absence of astrocytes. Therefore, the maintenance of high ADK expression levels in neurons of the adult CB may replace roles of ADK normally linked to astrocytes of the adult cerebrum and may provide needed support for maintenance of adenosine metabolism in the absence of astrocytes, and at the same time support high-energy demand and cell signaling.

## Synthesis

Reviewing Editor: Christophe Bernard, INSERM & Institut de Neurosciences des Systèmes Decisions are customarily a result of the Reviewing Editor and the peer reviewers coming together and discussing their recommendations until a consensus is reached. When revisions are invited, a fact-based synthesis statement explaining their decision and outlining what is needed to prepare a revision will be listed below. The following reviewer(s) agreed to reveal their identity: Hechen Bao, Mark Wall.

After a consultation, we agreed that the paper provides interesting novel information.

There are some relatively minor issues that need to be addressed. In particular the lack of quantification. It is OK to compare two images without quantification, provided that the conditions to obtain the images were exactly identical. But in general, some quantification is preferred.

Also, it would be nice to used estimation statistics, reporting effect size, and using Cumming representations instead of histograms.

You can read my editorial:

https://www.eneuro.org/content/6/4/ENEURO.0259-19.2019

and the commentary:

https://www.eneuro.org/content/6/4/ENEURO.0205-19.2019

Specific comments:

1. Throughout the results, the authors used the terms of “ADK-S” and “ADK-L” multiple times, but their definition is very unclear. For example, in line 208-210 and fig.1A, they mentioned “ubiquitous expression of ADK throughout the cerebrum with a uniform light staining indicative of ADK-S expressed in astrocyte processes and dark puncta reflecting the expression of ADK-L in nuclei (Fig 1A)”. This is confusing and lack of rigorous proof. Same issue for line 246-248.

In addition, they claimed ADK-S expressed in astrocyte processes (line 208) but lacks astrocyte marker staining.

2.Regarding ADK expression changes during brain development, it is curious how ADK-L/ADK-S ratio changes from P0-P15 in cerebrum? Also, in line 225-226, it is confusing how the authors define “maintained at high levels", because from the cerebellum plots (fig.2C), they also seem to significantly decreased from P0-adult.

3.In fig.2A,B, they used different housekeeping gene as reference, GAPDH for A, and b-tubulin for B. Is there any reasons for that?

4.The authors made their statements mainly based on observation rather than quantification in fig.3,4. It is hard to accept without performing statistic tests. The fig.4E also has very different background from the others. So some standardization and quantification are necessary to draw conclusions from those images.

5.Fig.5 is not clear to tell the colocalization at single cell level. The representative images are not convincing. In addition, the size of ROI/scale bar of those images are inconsistent, which makes the results harder to compare.

6.What is the control in fig.5N?

7.The statement in line 369-370, “The vast majority of IGL neurons are ADK/NeuN positive (Fig 7F)” lacks quantification.

## References

[B1] Atterbury A, Wall MJ (2009) Adenosine signalling at immature parallel fibre–Purkinje cell synapses in rat cerebellum. J Physiol 587:4497–4508. 10.1113/jphysiol.2009.176420 19651764 PMC2766653

[B2] Azevedo FA, Carvalho LR, Grinberg LT, Farfel JM, Ferretti RE, Leite RE, Jacob Filho W, Lent R, Herculano-Houzel S (2009) Equal numbers of neuronal and nonneuronal cells make the human brain an isometrically scaled-up primate brain. J Comp Neurol 513:532–541. 10.1002/cne.21974 19226510

[B3] Behesti H, Marino S (2009) Cerebellar granule cells: insights into proliferation, differentiation, and role in medulloblastoma pathogenesis. Int J Biochem Cell Biol 41:435–445. 10.1016/j.biocel.2008.06.017 18755286

[B4] Bjursell MK, Blom HJ, Cayuela JA, Engvall ML, Lesko N, Balasubramaniam S, Brandberg G, Halldin M, Falkenberg M, Jakobs C, Smith D, Struys E, von Döbeln U, Gustafsson CM, Lundeberg J, Wedell A (2011) Adenosine kinase deficiency disrupts the methionine cycle and causes hypermethioninemia, encephalopathy, and abnormal liver function. Am J Hum Genet 89:507–515. 10.1016/j.ajhg.2011.09.004 21963049 PMC3188832

[B5] Boison D (2008) The adenosine kinase hypothesis of epileptogenesis. Prog Neurobiol 84:249–262. 10.1016/j.pneurobio.2007.12.002 18249058 PMC2278041

[B6] Boison D (2009) Adenosine-based modulation of brain activity. Curr Neuropharmacol 7:158–159. 10.2174/157015909789152173 20190958 PMC2769000

[B7] Boison D (2012) Adenosine dysfunction in epilepsy. Glia 60:1234–1243. 10.1002/glia.22285 22700220 PMC3376389

[B8] Boison D (2013) Adenosine kinase: exploitation for therapeutic gain. Pharmacol Rev 65:906–943. 10.1124/pr.112.006361 23592612 PMC3698936

[B9] Boison D, Aronica E (2015) Comorbidities in neurology: is adenosine the common link? Neuropharmacology 97:18–34. 10.1016/j.neuropharm.2015.04.031 25979489 PMC4537378

[B10] Boison D, Yegutkin GG (2019) Adenosine metabolism: emerging concepts for cancer therapy. Cancer Cell 36:582–596. 10.1016/j.ccell.2019.10.007 31821783 PMC7224341

[B11] Boison D, Scheurer L, Zumsteg V, Rülicke T, Litynski P, Fowler B, Brandner S, Mohler H (2002) Neonatal hepatic steatosis by disruption of the adenosine kinase gene. Proc Natl Acad Sci USA 99:6985–6990. 10.1073/pnas.092642899 11997462 PMC124515

[B12] Boison D, Singer P, Shen HY, Feldon J, Yee BK (2012) Adenosine hypothesis of schizophrenia - opportunities for pharmacotherapy. Neuropharmacology 62:1527–1543. 10.1016/j.neuropharm.2011.01.048 21315743 PMC3119785

[B13] Braas KM, Newby AC, Wilson VS, Snyder SH (1986) Adenosine-containing neurons in the brain localized by immunocytochemistry. J Neurosci 6:1952–1961. 10.1523/JNEUROSCI.06-07-01952.1986 2426424 PMC6568579

[B14] Brown AM, Arancillo M, Lin T, Catt DR, Zhou J, Lackey EP, Stay TL, Zuo Z, White JJ, Sillitoe RV (2019) Molecular layer interneurons shape the spike activity of cerebellar Purkinje cells. Sci Rep 9:1742. 10.1038/s41598-018-38264-1 30742002 PMC6370775

[B15] Carletti B, Rossi F (2008) Neurogenesis in the cerebellum. Neuroscientist 14:91–100. 10.1177/1073858407304629 17911211

[B16] Crews FT, Mdzinarishvili A, Kim D, He J, Nixon K (2006) Neurogenesis in adolescent brain is potently inhibited by ethanol. Neuroscience 137:437–445. 10.1016/j.neuroscience.2005.08.090 16289890

[B17] DiCicco-Bloom E, Obiorah M (2017) Neural development and neurogenesis. In: Kaplan & Sadock’s comprehensive textbook of psychiatry (Sadock BJ, Sadock VA, Ruiz P, eds), Ed 10, pp 39–61. Philadelphia: Wolters Kluwer.

[B18] Etherington LA, Patterson GE, Meechan L, Boison D, Irving AJ, Dale N, Frenguelli B (2009) Astrocytic adenosine kinase regulates basal synaptic adenosine levels and seizure activity but not activity-dependent adenosine release in the hippocampus. Neuropharmacology 56:429–437. 10.1016/j.neuropharm.2008.09.016 18957298 PMC9972962

[B19] Gebril HM, Rose RM, Gesese R, Emond MP, Huo Y, Aronica E, Boison D (2020) Adenosine kinase inhibition promotes proliferation of neural stem cells after traumatic brain injury. Brain Commun 2:fcaa017. 10.1093/braincomms/fcaa017 32322821 PMC7158236

[B20] Goodman RR, Kuhar MJ, Hester L, Snyder SH (1983) Adenosine receptors: autoradiographic evidence for their location on axon terminals of excitatory neurons. Science 220:967. 10.1126/science.6302841 6302841

[B21] Gouder N, Scheurer L, Fritschy JM, Boison D (2004) Overexpression of adenosine kinase in epileptic hippocampus contributes to epileptogenesis. J Neurosci 24:692–701. 10.1523/JNEUROSCI.4781-03.2004 14736855 PMC6729249

[B22] Kiese K, Jablonski J, Boison D, Kobow K (2016) Dynamic regulation of the adenosine kinase gene during early postnatal brain development and maturation. Front Mol Neurosci 9:99. 10.3389/fnmol.2016.00099 27812320 PMC5071315

[B23] Kocsis JD, Eng DL, Bhisitkul RB (1984) Adenosine selectively blocks parallel-fiber-mediated synaptic potentials in rat cerebellar cortex. Proc Natl Acad Sci USA 81:6531–6534. 10.1073/pnas.81.20.6531 6093104 PMC391958

[B24] König N, Marty R (1981) Early neurogenesis and synaptogenesis in cerebral cortex. Bibl Anat (19):152–160. 7225063

[B25] Masino SA, Kawamura M Jr, Cote JL, Williams RB, Ruskin DN (2013) Adenosine and autism: a spectrum of opportunities. Neuropharmacology 68:116–121. 10.1016/j.neuropharm.2012.08.013 22940000 PMC3529135

[B26] Moffatt BA, Stevens YY, Allen MS, Snider JD, Pereira LA, Todorova MI, Summers PS, Weretilnyk EA, Martin-McCaffrey L, Wagner C (2002) Adenosine kinase deficiency is associated with developmental abnormalities and reduced transmethylation. Plant Physiol 128:812–821. 10.1104/pp.010880 11891238 PMC152195

[B27] Nalivaeva NN, Turner AJ, Zhuravin IA (2018) Role of prenatal hypoxia in brain development, cognitive functions, and neurodegeneration. Front Neurosci 12:825–825. 10.3389/fnins.2018.00825 30510498 PMC6254649

[B28] Namba K, Suzuki T, Nakata H (2010) Immunogold electron microscopic evidence of in situ formation of homo- and heteromeric purinergic adenosine A1 and P2Y2 receptors in rat brain. BMC Res Notes 3:323. 10.1186/1756-0500-3-323 21114816 PMC3009664

[B72] Nicot A, Lelièvre V, Tam J, Waschek JA, DiCicco-Bloom E (2002) Pituitary adenylate cyclase-activating polypeptide and sonic hedgehog interact to control cerebellar granule precursor cell proliferation. J Neurosci 22:9244–9254. 12417650 10.1523/JNEUROSCI.22-21-09244.2002 PMC6758018

[B29] Noctor SC, Flint AC, Weissman TA, Wong WS, Clinton BK, Kriegstein AR (2002) Dividing precursor cells of the embryonic cortical ventricular zone have morphological and molecular characteristics of radial glia. 22:3161–3173. 11943818 10.1523/JNEUROSCI.22-08-03161.2002 PMC6757532

[B30] Rakic P (2007) The radial edifice of cortical architecture: from neuronal silhouettes to genetic engineering. Brain Res Rev 55:204–219. 10.1016/j.brainresrev.2007.02.010 17467805 PMC2203611

[B31] Rossman IT, DiCicco-Bloom E (2008) Engrailed2 and cerebellar development in the pathogenesis of autism spectrum disorders. In: Autism: current theories and evidence (Zimmerman AW, ed), pp 3–40. Totowa: Humana Press.

[B70] Rossman IT, Lin L, Morgan KM, Digiovine M, Van Buskirk EK, Kamdar S, Millonig JH, Dicicco-Bloom E (2014) Engrailed2 modulates cerebellar granule neuron precursor proliferation, differentiation and insulin-like growth factor 1 signaling during postnatal development. Mol Autism 5:9. 10.1186/2040-2392-5-9 PMC3932947 24507165

[B32] Schmahmann JD (2004) Disorders of the cerebellum: ataxia, dysmetria of thought, and the cerebellar cognitive affective syndrome. J Neuropsychiatry Clin Neurosci 16:367–378. 10.1176/jnp.16.3.367 15377747

[B33] Shen Q, Wang Y, Dimos JT, Fasano CA, Phoenix TN, Lemischka IR, Ivanova NB, Stifani S, Morrisey EE, Temple S (2006) The timing of cortical neurogenesis is encoded within lineages of individual progenitor cells. Nat Neurosci 9:743–751. 10.1038/nn1694 16680166

[B34] Silva L, Plösch T, Toledo F, Faas MM, Sobrevia L (2020) Adenosine kinase and cardiovascular fetal programming in gestational diabetes mellitus. Biochim Biophys Acta 1866:165397. 10.1016/j.bbadis.2019.01.023 30699363

[B35] Staufner C, Lindner M, Dionisi-Vici C, Freisinger P, Dobbelaere D, Douillard C, Makhseed N, Straub BK, Kahrizi K, Ballhausen D, la Marca G, Kölker S, Haas D, Hoffmann GF, Grünert SC, Blom HJ (2016) Adenosine kinase deficiency: expanding the clinical spectrum and evaluating therapeutic options. J Inherit Metab Dis 39:273–283. 10.1007/s10545-015-9904-y 26642971

[B36] Studer FE, Fedele DE, Marowsky A, Schwerdel C, Wernli K, Vogt K, Fritschy JM, Boison D (2006) Shift of adenosine kinase expression from neurons to astrocytes during postnatal development suggests dual functionality of the enzyme. Neuroscience 142:125–137. 10.1016/j.neuroscience.2006.06.016 16859834

[B37] Susan S, Harold E, Jeremiah CH (2008) Gray’s anatomy, Ed 40. Spain: Churchill Livingstone.

[B38] ten Donkelaar HJ, Lammens M, Wesseling P, Thijssen HO, Renier WO (2003) Development and developmental disorders of the human cerebellum. J Neurol 250:1025–1036. 10.1007/s00415-003-0199-9 14504962

[B71] Tao Y, Black IB, DiCicco-Bloom E (1996) Neurogenesis in neonatal rat brain is regulated by peripheral injection of basic fibroblast growth factor (bFGF). J Comp Neurol 376:653–663. 8978476 10.1002/(SICI)1096-9861(19961223)376:4<653::AID-CNE11>3.0.CO;2-N

[B39] Wagner MJ, Kim TH, Savall J, Schnitzer MJ, Luo L (2017) Cerebellar granule cells encode the expectation of reward. Nature 544:96–100. 10.1038/nature21726 28321129 PMC5532014

[B40] Wall MJ, Atterbury A, Dale N (2007) Control of basal extracellular adenosine concentration in rat cerebellum. J Physiol 582:137–151. 10.1113/jphysiol.2007.132050 17446223 PMC2075308

[B41] Zhang L, Goldman JE (1996) Generation of cerebellar interneurons from dividing progenitors in white matter. Neuron 16:47–54. 10.1016/S0896-6273(00)80022-7 8562089

[B42] Zhang X, Jia D, Liu H, Zhu N, Zhang W, Feng J, Yin J, Hao B, Cui D, Deng Y, Xie D, He L, Li B (2013) Identification of 5-Iodotubercidin as a genotoxic drug with anti-cancer potential. PLoS One 8:e62527. 10.1371/journal.pone.0062527 23667485 PMC3646850

[B43] Zhang Z-w (2004) Maturation of layer V pyramidal neurons in the rat prefrontal cortex: intrinsic properties and synaptic function. J Neurophysiol 91:1171–1182. 10.1152/jn.00855.2003 14602839

